# Differences in determinants of active aging between older Brazilian and English adults: ELSI-Brazil and ELSA

**DOI:** 10.1590/0102-311XEN076823

**Published:** 2023-10-13

**Authors:** Janderson Diego Pimenta da Silva, Isadora Viegas Martins, Luciana Helena Reis Braga, Cesar Messias de Oliveira, Maria Fernanda Lima-Costa, Luciana de Souza Braga, Juliana Lustosa Torres

**Affiliations:** 1 Universidade Federal de Minas Gerais, Belo Horizonte, Brasil.; 2 University College London, London, U.K.; 3 Instituto René Rachou, Fundação Oswaldo Cruz, Belo Horizonte, Brasil.

**Keywords:** Healthy Aging, Aging, Population Dynamics, Health Promotion, Health of the Elderly, Envelhecimento Saudável, Envelhecimento, Dinâmica Populacional, Promoção da Saúde, Saúde do Idoso, Envejecimiento Saludable, Envejecimiento, Dinámica Poblacional, Promoción de la Salud, Salud del Anciano

## Abstract

This study aimed to investigate differences in determinants of active aging between older Brazilian and English adults and to verify the association of behavioral, personal, and social determinants with physical health. This cross-sectional study was based on the ELSI-Brazil (2015-2016) and ELSA (2016-2017) cohorts. Active aging determinants included behavior (smoking, sedentary lifestyle, and poor sleep quality), personal (cognitive function and life satisfaction), and social determinants (education, loneliness, and volunteering), according to the World Health Organization. Physical health included activities limitation and multimorbidity. We estimated age- and sex-adjusted prevalence for each indicator and mean score, and used the negative binomial regression for statistical analysis. We included 16,642 participants, 9,409 from Brazil and 7,233 from England. Overall, all active aging determinants were worse in Brazil than in England, except for life satisfaction (no difference). The most remarkable difference was found for social determinants score in Brazil (mean difference of 0.18; p < 0.05), mainly due to a significantly lower education level in Brazil (70.6%; 95% confidence interval - 95%CI: 69.7-71.5) than England (37.1%; 95%CI: 35.1-39.1). All determinants (behavioral, personal, and social) were associated with health in Brazil and in England. However, the behavioral domain was stronger associated with health in England (coefficient = 2.76; 95%CI: 2.46-3.10) than in Brazil (coefficient = 1.38; 95%CI: 1.26-1.50; p < 0.001). Older English adults beneficiate more from healthier behaviors than Brazilians, which depend more on social policies.

## Resumo

Este estudo transversal objetivou investigar as diferenças nos determinantes do envelhecimento ativo entre idosos brasileiros e ingleses, e verificar a associação de determinantes comportamentais, pessoais e sociais com a saúde física. A pesquisa baseou-se nas coortes ELSI-Brasil (2015-2016) e ELSA (2016-2017). Os determinantes do envelhecimento ativo incluíram os determinantes comportamentais (tabagismo, sedentarismo e má qualidade do sono), pessoais (função cognitiva e satisfação com a vida) e sociais (educação, solidão e voluntariado), de acordo com a Organização Mundial da Saúde. A saúde física incluiu limitação de atividades e multimorbidade. Prevalências ajustadas por idade e sexo foram calculadas para cada indicador e escores médios, utilizando-se a regressão binomial negativa para a análise estatística. A pesquisa incluiu 16.642 participantes, sendo 9.409 do Brasil e 7.233 da Inglaterra. No geral, todos os determinantes do envelhecimento ativo foram piores no Brasil do que na Inglaterra, exceto a satisfação com a vida (sem diferença). A diferença mais marcante refere-se ao escore de determinantes sociais no Brasil (diferença média de 0,18; p < 0,05), principalmente devido à escolaridade significativamente menor no Brasil (70,6%; intervalo de 95% de confiança - IC95%: 69,7-71,5) do que na Inglaterra (37,1%; IC95%: 35,1-39,1). Todos os determinantes (comportamentais, pessoais e sociais) estiveram associados à saúde no Brasil e na Inglaterra. No entanto, o domínio comportamental foi mais fortemente associado à saúde na Inglaterra (coeficiente = 2,76; IC95%: 2,46-3,10) do que no Brasil (coeficiente = 1,38; IC95%: 1,26-1,50) (p < 0,001). Idosos ingleses se beneficiam mais de comportamentos mais saudáveis do que os brasileiros, que dependem mais de políticas sociais.

## Resumen

Este estudio transversal tuvo como objetivo investigar las diferencias en los determinantes del envejecimiento activo entre personas mayores brasileñas e inglesas, y verificar la asociación de determinantes conductuales, personales y sociales con la salud física. La investigación se basó en las cohortes ELSI-Brasil (2015-2016) y ELSA (2016-2017). Los determinantes del envejecimiento activo incluyeron determinantes conductuales (tabaquismo, sedentarismo y mala calidad del sueño), personales (función cognitiva y satisfacción con la vida) y sociales (educación, soledad y voluntariado), según la Organización Mundial de la Salud. La salud física incluyó la limitación de actividades y la multimorbilidad. Se calcularon las prevalencias ajustadas por edad y sexo para cada indicador y los puntajes medios, usando la regresión binomial negativa para el análisis estadístico. La encuesta incluyó a 16.642 participantes, 9.409 de Brasil y 7.233 de Inglaterra. En general, todos los determinantes del envejecimiento activo fueron peores en Brasil que en Inglaterra, salvo la satisfacción con la vida (sin diferencia). La diferencia más llamativa se refiere al puntaje de los determinantes sociales en Brasil (diferencia media de 0,18; p < 0,05), sobre todo debido al nivel educativo significativamente más bajo en Brasil (70,6%; intervalo de 95% de confianza - IC95%: 69,7-71,5) que en Inglaterra (37,1%; IC95%: 35,1-39,1). Todos los determinantes (conductuales, personales y sociales) se asociaron con la salud en Brasil y en Inglaterra. Sin embargo, el dominio conductual se asoció más fuertemente con la salud en Inglaterra (coeficiente = 2,76; IC 95% 2,46-3,10) que en Brasil (coeficiente = 1,38; IC95%: 1,26-1,50) (p < 0,001). Las personas mayores inglesas se benefician más de comportamientos más saludables que los brasileños, que dependen más de las políticas sociales.

## Introduction

Population has challenged global health due to a steep and unequal older adult population growth. Globally, the population aged 60 years and over is expected to increase from 202 million in 1950 to 3.1 billion in 2100, representing an absolute growth of 15.2 times ^1^. As a result, direct impacts on social, economic, and health aspects have emerged ^2^. Population aging is associated with a higher prevalence of chronic diseases and a greater number of individuals living with an accumulation of two or more chronic diseases, a condition defined as multimorbidity ^3^. Multimorbidity has been associated with mortality, lower quality of life ^4^, and poor functional ability ^5^. Therefore, policies towards promoting active and healthy aging are essential.

Earlier in the 2000s, the World Health Organization (WHO) proposed the *Policy Framework on Active Ageing*, guiding policy initiatives that enhance the involvement of older adults in society and promote healthy and active aging. Active aging has been defined as “*the process of optimizing opportunities for health, lifelong learning, participation, and security in order to enhance the quality of life as people age*” ^6^ (p. 44).

According to the WHO, a set of interrelated factors, the determinants of active aging, may shape whether a person ages actively over the life course. These determinants include behavioral (i.e., smoking, healthy eating, physical activity, sleep) and personal determinants (i.e., biological, psychological, and genetic factors) that are individually defined but reflect other macro determinants, including the physical environment (i.e., urban planning and housing); social determinants (i.e., education, social isolation, and loneliness); economic determinants (i.e., socioeconomic status and employment conditions); and health and social services, fundamental to promote health with fewer disabilities associated with chronic diseases and reduce health inequalities. Culture and gender can be seen as cross-cutting determinants that shape people and their environment. The determinants of active aging might be shaped by public policies developed by strategic actions based on four key pillars: health, lifelong learning, participation, and security ^6^.

However, studies have not explored in depth the concept of active aging and the literature present no theoretical consensus on it. A Spanish study evaluated the effect of active aging pillars on life expectancy and found a significant association only with the physical component of the health pillar ^7^. In China, the health and security pillars were strongly linked to participation ^8^. In Brazil, nationally representative analyses, based on the *National Health Survey* (PNS) conducted in 2013, found that active aging, defined in social, physical, intellectual, and work activity dimensions, demonstrated disparities between socioeconomic status, race, sex, and age ^9^ ^,^ ^10^. Moreover, a local study conducted in Uberaba city (Minas Gerais State, Brazil) with 957 community-dwelling older adults proposed a structural active aging model and found that the most relevant determinant category was the health/social services determinants, followed by the social determinants ^11^.

To the best of our knowledge, high- and middle-income cross-country comparative studies on active aging determinants among older adults are scarce. Most of the evidence available has focused on survival, birth cohorts ^12^, and healthy aging scores ^13^ ^,^ ^14^, but not the determinants of active aging. In 2020, the population aged 65 years and over comprised 9.8% of the total population in Brazil ^15^ and 21% in England ^16^. Both countries present universal public health systems that are primary-care oriented, named the Brazilian Unified National Health System (SUS, since 1988) and the National Health System (NHS) in England (since 1948). Moreover, England is a high-income country with one of the best Human Development Index globally, which indicates the best scenario to deal with the aging process. On the other hand, Brazil is an upper-middle-income country with a faster aging process than England, presenting several vulnerabilities in dealing with the aging process, including its continental characteristic, large population, and persistent socioeconomic inequalities. Therefore, this study aimed to investigate differences in determinants of active aging between older Brazilian and English adults, and to verify the association of behavioral, personal, and social determinants with the physical component of health, using two harmonized nationally representative cohorts i.e., the *Brazilian Longitudinal Study of Aging* (ELSI-Brazil) and the *English Longitudinal Study of Ageing* (ELSA).

## Method

### Study population

Cross-sectional data from ELSI-Brazil and ELSA cohorts were used. ELSI-Brazil is a population-based household longitudinal study with community-dwelling older adults aged 50 years and older, living across 70 cities in Brazil. Sampling procedures were based on 3-stage geographic stratification according to the population size of the 70 municipalities included, distributed across the five great regions of Brazil, accounting for 9,412 interviews. Baseline data used in the current analysis were collected in 2015-2016. Further information on ELSI-Brazil can be found on the ELSI-Brazil homepage (http://elsi.cpqrr.fiocruz.br/) and in a previous publication. ELSI-Brazil was approved by the Research Ethics Committee from the René Rachou Institute of the Oswaldo Cruz Foundation (protocol number 34649814.3.0000.5091).

ELSA is a population-based household cohort with community-dwelling older adults aged 50 years and older living in England that started in 2002-2003 with biannual interviews. Sampling selection included participants of the *Health Survey for England* (HSE - 1998, 1999, and 2001), a household representative survey with a multi-stage stratified sample design using postcodes sectors as primary sample units. For the current study, data from wave 8 was used, comprising 8,445 participants (7,223 core participants), collected in 2016-2017, the closest calendar year to ELSI-Brazil baseline data. Further information on ELSA can be found on the ELSA homepage (https://www.elsa-project.ac.uk/) and in a previous publication. ELSA was approved by London Multicentre Research Ethics Committee (protocol number MREC/01/2/91).

### Active aging determinants

A total of three groups of active aging determinants were included due to harmonized data availability between the two cohorts:

(a) Behavioral determinants: smoking status, considering the current self-report smoking status (yes or no); physical inactivity, defined as practicing vigorous or moderate physical activity less than once per week (yes or no); and poor sleep quality, defined by self-report on poor or very poor sleep quality (yes or no);

(b) Personal determinants: verbal fluency by naming as many different animals as possible in one minute (continuous) was used to asses cognitive function; and a ten-point scale (continuous) was used to rate overall life satisfaction;

(c) Social determinants: level of education is categorized in complete years of schooling, according to formal education in each country (lower, intermediate, or higher). In Brazil, formal education was organized into incomplete first level (0-7 schooling years), complete first level up to incomplete second level (8-11 schooling years), and complete second level or higher (≥ 12 schooling years). In England, the 3-way education division is qualified to a level lower than “O level” or equivalent (typically 0-11 years of schooling), qualified to a level lower than “A level” or equivalent (typically 12-13 years of schooling), and a higher qualification (typically > 13 years of schooling); loneliness, assessed by the question single-item direct measure “how often do you feel alone/lonely?” (never, some of the time, or often); and volunteering, considering its self-reported frequency (at least once a week, at least once a month, or never/not very often).

### Physical health

To achieve active aging, healthcare and support systems aim to prevent chronic diseases and disabilities, which are related to the physical component of health. Thus, two indicators of health were included: activities of daily living (ADL) limitation, measured by participants’ self-report of difficulties in walking, transferring, toileting, bathing, dressing, or eating using the modified Katz index (0 or 1+ activities); and multimorbidity, considering previous medical diagnosis for cardiovascular diseases (hypertension, stroke, heart attack, angina or heart failure), high cholesterol, neurological diseases (Parkinson’s or Alzheimer’s diseases), chronic lung disease, diabetes, arthritis or rheumatism, asthma, and cancer (0-1 or 2+ chronic conditions).

### Statistical analyses

Firstly, age- and sex-adjusted prevalence was calculated for each indicator, by country, using the standard population at the individual level (directly standardized method) to compare the prevalence rates between Brazil and England. Moreover, the unadjusted prevalence was also calculated accounting for complex sampling designs. Secondly, raw scores were elaborated for each group of active aging determinants and health by summing the indicators. All indicators were arranged to create scores ranging from 0 to 1, in which higher scores indicate worse performance. Then, since the health score showed overdispersion, negative binomial regression models were used to estimate its association with behavioral, personal, and social determinants. The models were stratified by country and then jointed with an interaction term with country to test differences by country. Finally, the predicted health score was plotted according to the statistically significant determinants.

All analyses were performed using Stata software, version 17.0 (https://www.stata.com), and accounted for the survey weights.

## Results

This study included 9,299 older Brazilian adults and 7,233 older English adults, accounting for 16,522 participants. From ELSI-Brazil, 113 participants were not included due to missing data on physical health indicators, and 1,212 participants from ELSA were not included since they were not core members (i.e., have no sampling weight assigned). Table 1 shows the age- and sex-adjusted prevalence of active aging determinants by country. Overall, all determinants were worse in Brazil than in England, except for life satisfaction, a personal determinant with a similar mean pattern in both countries. Within the behavioral determinants, the highest difference was found for poor sleep quality (20.6%): in Brazil, the prevalence was 45.4% (95% confidence interval - 95%CI: 44.3-46.5), whereas in England it was 24.8% (95%CI: 22.4-27.2). Considering the social determinants, the highest difference between countries was found for the lower education level, 70.6% (95%CI: 69.7-71.5) among older Brazilian adults and 37.1% (95%CI: 35.1-39.1) among the English counterparts. Regarding health, Brazil showed 46.7% (95%CI: 45.6-47.8) of multimorbidity and England, 38.6% (95%CI: 36.4-40.7).

**Table 1:** Age- and sex-adjusted prevalence of active aging determinants and health among older Brazilians and English adults. *Brazilian Longitudinal Study of Aging* (ELSI-Brazil, 2015-2016) and *English Longitudinal Study of Ageing* (ELSA, 2016-2017).

	Brazil [n = 9,299]	England [n = 7,223]	Difference [Brazil - England]
% (95%CI) *	% (95%CI) *	%
Behavior determinants			
Smoking	15.9 (15.2-16.7)	10.9 (9.2-12.5)	+5.0 **
Physical inactivity	40.3 (39.2-41.3)	23.1 (21.5-24.8)	+17.2 **
Poor sleep quality	45.4 (44.3-46.5)	24.8 (22.4-27.2)	+20.6 **
Personal determinants			
Mean cognitive function	11.1 (11.1-11.2)	21.8 (21.6-22.1)	-10.7 **
Mean life satisfaction	7.2 (7.1-7.2)	7.3 (7.1-7.4)	0.1
Social determinants			
Education			
Lower	70.6 (69.7-71.5)	37.1 (35.1-39.1)	+33.5 **
Intermediate	9.9 (9.3-10.5)	29.5 (27.1-31.9)	-19.6 **
Higher	19.5 (18.7-20.3)	33.4 (30.9-35.9)	-13.9 **
Loneliness			
Never	51.8 (50.7-52.9)	70.9 (68.3-73.4)	-19.1 **
Some of the time	30.6 (29.6-31.7)	23.0 (20.5-25.5)	+7.6 **
Often	17.6 (16.7-18.5)	6.1 (5.4-6.9)	+11.5 **
Volunteering			
At least once a week	7.3 (6.7-7.8)	17.5 (15.4-19.7)	-10.2 **
At least once a month	4.4 (3.9-4.8)	10.3 (9.0-11.6)	-5.9 **
Never or not very often	88.4 (87.7-89.0)	72.2 (69.7-74.7)	+16.2 **
Physical health			
ADL limitation ***	18.5 (17.7-19.4)	16.3 (15.4-17.2)	+2.2 **
Multimorbidity ^#^	46.7 (45.6-47.8)	38.6 (36.4-40.7)	+8.1 **

95%CI: 95% confidence interval; ADL: activities of daily living.

* Age- and sex-adjusted prevalence based on the directly standardized method;

** Values without overlapping 95%CI;

*** Difficulties in walking, transferring, toileting, bathing, dressing, or eating;

^#^ Considering cardiovascular diseases (hypertension, stroke, heart attack, angina, or heart failure), high cholesterol, neurological diseases (Parkinson’s or Alzheimer’s diseases), chronic lung disease, diabetes, arthritis or rheumatism, asthma, and cancer.

Table 2 shows scores calculated for each active aging determinant and health. Again, all scores were worse in Brazil, with a higher mean difference for social determinant score (0.18): 0.66 (95%CI: 0.65-0.66) in Brazil and 0.48 (95%CI: 0.47-0.49) in England, followed by behavioral and personal (0.15). 

**Table 2:** Age- and sex-adjusted active aging determinant scores * and health among older Brazilians and English adults. *Brazilian Longitudinal Study of Aging* (ELSI-Brazil, 2015-2016) and *English Longitudinal Study of Ageing* (ELSA, 2016-2017).

	Brazil [N = 9,299]	England [N = 7,223]	Difference [Brazil - England]
	Mean (95%CI) **	Mean (95%CI) **	
Behavioral determinants score ***	0.34 (0.33-0.34)	0.19 (0.18-0.20)	+0.15 ^#^
Personal determinants score ^##^	0.51 (0.51-0.52)	0.36 (0.36-0.37)	+0.15 ^#^
Social determinants score ^###^	0.66 (0.65-0.66)	0.48 (0.47-0.49)	+0.18 ^#^
Health score ^§^	0.24 (0.23-0.24)	0.20 (0.19-0.21)	+0.04 ^#^

95%CI: 95% confidence interval.

* Higher scores represent worse determinants;

** Age- and sex-adjusted mean based on the directly standardized method;

*** Smoking, physical inactivity, and poor sleep quality;

^#^ Values without overlapping 95%CI;

^##^ Cognitive function and life satisfaction;

^###^ Education, loneliness, and volunteering;

^§^ A activities of daily living limitation and multimorbidity.

The adjusted association between health score and the active aging scores are presented in Table 3. As expected, in both countries, the behavioral, personal, and social determinant scores were associated with health score. In Brazil, the social determinant score showed the highest force of association, indicating a 70% increase in health score every 0.1 increase in the social determinant score. Differently, the behavioral determinant score showed the highest force of association in England, indicating a 176% increase in health score every 0.1 increase in the behavioral determinant score. The interaction terms between country and the active aging determinant revealed that the behavioral determinant (p < 0.001) was the only active aging determinant that was different between Brazil and England, with a stronger association in England.

**Table 3:** Adjusted models for the association between active aging determinants * and health score ** among older Brazilians and English adults. *Brazilian Longitudinal Study of Aging* (ELSI-Brazil, 2015-2016) and *English Longitudinal Study of Ageing* (ELSA, 2016-2017).

	Brazil [N = 8,179]		England [N = 5,906]		p-value for the interaction term
	Coefficient	95%CI	Coefficient	95%CI		
Age	1.01	1.01-1.02	1.03	1.02-1.03	-
Sex					
Male	1.00		1.00		
Female	1.28	1.22-1.34	0.94	0.89-0.99	-
Behavioral determinants score ***	1.38	1.26-1.50	2.76	2.46-3.10	< 0.001
Personal determinants score ^#^	1.29	1.15-1.46	1.56	1.34-1.83	0.115
Social determinants score ^##^	1.70	1.53-1.90	1.34	1.17-1.54	0.814

95%CI: 95% confidence interval.

Note: coefficients based on negative binomial regression models.

* Higher scores represent worse determinants;

** Based on activities of daily living limitation and number of chronic conditions;

*** Including smoking, physical inactivity, and poor sleep quality;

^#^ Including cognitive function and life satisfaction;

^##^ Including education, loneliness, and volunteering.

**Figure 1:**
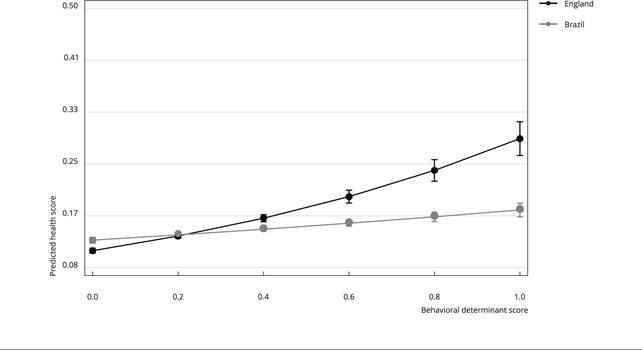
Predicted health score, according to behavioural determinant score and country. *Brazilian Longitudinal Study of Aging* (ELSI-Brazil, 2015-2016) and *English Longitudinal Study of Ageing* (ELSA, 2016-2017).

To obtain the behavioral and health association, which statistically differed by country, the expected health score by country was plotted according to the behavioral determinants score. In England, health score presents greater variation according to behavioral determinants score than in Brazil (Figure 1). For example, in the worst behavioral score, at one score point, the expected health score in England is 0.29 (95%CI: 0.26-0.32), whereas, in Brazil, this expected health score equals to 0.18 (95%CI: 0.17-0.19).

## Discussion

This study found that all age- and sex-adjusted prevalence of active aging determinants were worse in Brazil than in England, except for life satisfaction, an indicator of personal determinants found to be similar. However, when grouped into scores, personal determinants were also worse among older Brazilian adults, similar to the other scores. As expected, all determinants (behavioral, personal, and social) were associated with health in Brazil and England. However, the behavioral determinant was stronger associated with health score in England than in Brazil.

In general, the worst performance of active aging determinants in Brazil can be attributed to the lower availability of economic resources and the reduced welfare capacity to invest on health and educational policies. Although the economic resources have not been directly measured in the current study, the gross domestic product (GDP) in 2019 was USD 1.88 trillion in Brazil and USD 2.88 trillion in the United Kingdom according to the World Bank data ^17^. Moreover, despite presenting similar health expenditure regarding GDP (9.6% and 10.1%, respectively), the public health expenditure was significantly lower in Brazil: USD 606 per capita compared with USD 3,107 in the United Kingdom ^18^. One of the prerogatives of the 2030 United Nations Agenda is to significantly increase health financing and the recruitment, development, training, and retention of health personnel, especially in the most vulnerable territories ^18^. Alongside more investments, it is also necessary to target aging-friendly places to optimize opportunities for active aging by supportive environments for older adults in different areas (long-term care, built open spaces, transportation, social protection, and public policies changes) ^6^.

The worst education level in Brazil reflects past poorer policies in this area, characterized by unequal access to primary school from 1930s to at least 1950s. Ever since, more recent policies have increased the proportion of literate older adults, rising from 55.8% in 1991 to 77.7% in 2018 ^19^. However, their reflection will be seen in the subsequent aging cohorts since they only impact the younger birth cohorts. Low education presents deleterious effects that remain as people age ^20^ ^,^ ^21^, compromising access to health and better working conditions, and leading to higher levels of health risk behaviors. Our results demonstrated that weekly volunteering frequency is still very low in Brazil compared to England (7.3% vs. 17.5%, respectively), revealing important cultural differences. It is recommended to support civil societies in promoting volunteering, which is essential to foster lifelong learning6. Non-formal educational settings also develop learning abilities at all ages by improving access to information, promoting intergenerational exchanges, or providing training on aging.

Regarding behavioral determinants, we found worse age-and sex-adjusted prevalence in Brazil than in England, considering smoking, physical inactivity, and poor sleep quality. Our results are consistent with physical inactivity inequalities previously reported when comparing data from Latin American and Caribbean adults (39.1%) with Central and Eastern European countries ^22^ adults (23.4%). Our results are also consistent with reports that 80% of all current smokers live in low- and middle-income countries. Socioeconomic inequalities were also observed within countries towards more socially vulnerable individuals regarding smoking ^23^ and physical inactivity ^24^, possibly due to a lack of resources, social support, and motivation to modify harmful behaviors ^24^ and higher multimorbidity prevalence. However, behavioral determinants might be modifiable by public policies, and interventions encouraging better lifestyle choices have been reported with a 70% rate of success ^25^. These include adequate leisure public spaces, fostering empowerment, safety, and social inclusion.

Despite Brazil showing worse behavioral determinants than England, these were strongly associated with health in England, demonstrating that older English adults beneficiate more from healthier behaviors. Evidence in England has pointed that the combination of smoking and physical inactivity increases the multimorbidity onset in 135% over four years ^26^, and that poor sleep quality was associated with worse physical/mental health and cognitive function ^27^. Similar associations were also found in Brazil ^24^. However, health in Brazil depends more on other factors, such as social determinants. For example, worse health was more associated with lower-educated individuals ^14^, and, in Brazil, more people present lower education and live with disabilities ^28^. Low-educated older adults reported almost twice ADL limitation (≥ 2 limitations) when compared with high-educated older adults (40.8% vs. 22.5% in Brazil and 28.8% vs. 14.6% in England) ^29^. They also tend to live shorter due to poorer healthcare, security, income, and general life ^6^. Moreover, loneliness prevalence, which we found to be higher in Brazil, decreases the likelihood of healthy aging more among Brazilians ^30^.

Nevertheless, differences in health were not always reported. A Spanish study evaluated the effect of active aging pillars on life expectancy and found a significant association only with the physical component of the health pillar ^7^. These previous conflicting results corroborate the complexity of active aging framework and demonstrate different patterns between countries. The cumulative impact of lifelong social disparities on older adults from low- and middle-income countries might partially explain these results ^6^.

We also highlight that life satisfaction, a personal active aging determinant, was the only similar indicator between older Brazilians and older English adults. Life satisfaction is a subjective feeling related to income, personal characteristics, attitudes, relationships, age, birth cohort, and broader social environment ^31^. Gender and culture shape people and their lives, embedded with individual and context determinants. A perceived single indicator that captures such mixture may attenuate differences and explain the similar pattern of life satisfaction between older Brazilian and English adults found in this study.

Overall, our findings on determinants of active aging pointed that Brazil presented the worst determinant indicators for personal, behavioral, and social determinants, which leads to worse health. Therefore, efforts to enhance public health expenditure, universal coverage, quality of healthcare, and interventions encouraging healthier lifestyle choices are needed. Due to the strong relationship between the active aging determinants and health, public actions to achieve active aging must be intersectional and target citizenship.

This study presents some strengths and limitations that should be acknowledged. As for strengths, we point out the use of two large harmonized nationally representative datasets of older Brazilian and English adults, which allowed us to compare active aging determinants and health in both countries. Moreover, we used data from quite similar calendar years (2015-2016 in Brazil and 2016-2017 in England), embedding the interviews into similar world contexts. As limitations, we highlight that this is a cross-sectional study in which survival bias may play a distinct role in each country due to differences in life expectancy. Furthermore, we found difficulties in operationalizing the determinants of active aging and differentiating them from the key policy pillars due to overlapping definitions that can limit the interpretation of our results in terms of “active”. In this study, active aging determinants were only linked to objectives for health indicators, but it also included objectives for participation and security since they are complementary. Nevertheless, the results revealed insights into key determinants that need improvement to promote a better physical health profile.

In conclusion, this investigation revealed differences in active aging determinants in Brazil and England, demonstrating worse determinants in Brazil that may be attributable to lower availability of economic resources and the poorer education profile of the age group studied. Insights provided from the prevalence of active aging determinants may highlight areas that deserve more effective public policies in Brazil, such as educational and volunteering policies, and in England, such as encouraging better lifestyle choices and healthcare policies.

## References

[1] Department of Economic and Social Affairs, Population Division, United Nations World population prospects 2019.. https://population.un.org/wpp2019/Download/Standard/Population/.

[2] Andrade EIG, Cherchiglia ML, Souza PRB, Andrade FB, Mambrini JVM, Lima-Costa MF (2018). Factors associated with the receipt of pensions among older adults ELSI-Brazil. Rev Saúde Pública.

[3] Nunes BP, Batista SRR, Andrade FB, Souza PRBS, Lima-Costa MF, Facchini LA (2018). Multimorbidity the Brazilian Longitudinal Study of Ageing (ELSI-Brazil). Rev Saúde Pública.

[4] Rivera-Almaraz A, Manrique-Espinoza B, Ávila-Funes JA, Chatterji S, Naidoo N, Kowal P (2018). Disability, quality of life and all-cause mortality in older Mexican adults association with multimorbidity and frailty. BMC Geriatr.

[5] Schmidt TP, Wagner KJP, Schneider IJC, Danielewicz AL (2020). Padrões de multimorbidade e incapacidade funcional em idosos brasileiros estudo transversal com dados da Pesquisa Nacional de Saúde. Cad Saúde Pública.

[6] Centro Internacional de Longevidade Brasil (2015). Envelhecimento ativo: um marco político em resposta à revolução da longevidade.

[7] Hijas-Gómez A, Ayala A, Rodríguez-García M, Rodríguez-Blázquez C, Rodríguez-Rodríguez V, Rojo-Pérez F (2020). The WHO active ageing pillars and its association with survival findings from a population-based study in Spain. Arch Gerontol Geriatr.

[8] Yang Y, Meng Y, Dong P (2020). Health, security and participation a structural relationship modeling among the three pillars of active ageing in china. Int J Environ Res Public Health.

[9] Sousa NFS, Lima MG, Cesar CLG, Barros MBA (2018). Active aging prevalence and gender and age differences in a population-based study. Cad Saúde Pública.

[10] Sousa NFS, Medina LPB, Bastos TF, Monteiro CN, Lima MG, Barros MBA (2019). Social inequalities in the prevalence of indicators of active ageing in the Brazilian population: National Health Survey, 2013.. Rev Bras Epidemiol.

[11] Oliveira NGN, Tavares DMDS (2020). Active ageing among elderly community members structural equation modeling analysis. Rev Bras Enferm.

[12] Aida J, Cable N, Zaninotto P, Tsuboya T, Tsakos G, Matsuyama Y (2018). Social and behavioural determinants of the difference in survival among older adults in Japan and England. Gerontology.

[13] Fuente J, Caballero FF, Sánchez-Niubó A, Panagiotakos DB, Prina AM, Arndt H (2018). Determinants of health trajectories in England and the United States an approach to identify different patterns of healthy ageing. J Gerontol A Biol Sci Med Sci.

[14] Wu YT, Daskalopoulo C, Terrera GM, Niubo AS, Rodríguez-Artalejo F, Ayuso-Mateos JL (2020). Education and wealth inequalities in healthy ageing in eight harmonised cohorts in the ATHLOS consortium a population-based study. Lancet Public Health.

[15] Instituto Brasileiro de Geografia e Estatística Projeção da população do Brasil e das Unidades da Federação..

[16] Office for National Statistics Population estimates for the UK, England and Wales, Scotland and Northern Ireland: mid-2020..

[17] World Bank World development indicators. Popular indicators..

[18] Vieira FS (2020). Health financing in Brazil and the goals of the 2030 Agenda: high risk of failure.. Rev Saúde Pública.

[19] Travassos GF, Coelho AB, Arends-Kuenning MP (2020). The elderly in Brazil demographic transition, profile, and socioeconomic condition. Rev Bras Estud Popul.

[20] Giacomin KC, Duarte YAO, Camarano AA, Nunes DP, Fernandes D (2018). Care and functional disabilities in daily activities - ELSI-Brazil. Rev Saúde Pública.

[21] Wu YT, Daskalopoulou C, Terrera GM, Niubo AS, Rodríguez-Artalejo F, Ayuso-Mateos JL (2020). Education and wealth inequalities in healthy ageing in eight harmonised cohorts in the ATHLOS consortium a population-based study. Lancet Public Health.

[22] Guthold R, Stevens G, Riley L, Bull F (2018). Worldwide trends in insufficient physical activity from 2001 to 2016: a pooled analysis of 358 population-based surveys with 1·9 million participants.. Lancet Global Health.

[23] Malta D, Gomes C, Andrade F, Prates E, Alves F, Oliveira P (2021). Tobacco use, cessation, secondhand smoke and exposure to media about tobacco in Brazil: results of the National Health Survey 2013 and 2019.. Rev Bras Epidemiol.

[24] Peixoto SV, Mambrini JVM, Firmo JOA, Loyola AI, Junior PRBS, Andrade FB (2018). Physical activity practice among older adults: results of the ELSI-Brazil.. Rev Saúde Pública.

[25] Sánchez-González D, Rojo-Pérez F, Rodríguez-Rodríguez V, Fernández-Mayoralas G (2020). Environmental and psychosocial interventions in age-friendly communities and active ageing a systematic review. Int J Environ Res Public Health.

[26] Dhalwani NN, Zaccardi F, O'Donovan G.Carter P.Hamer M.Yates T (2017). Association between lifestyle factors and the incidence of multimorbidity in an older english population. J Gerontol A Biol Sci Med Sci.

[27] Gadie A, Shafto M, Leng Y, Kievit RA, Cam- CAN (2017). How are age-related differences in sleep quality associated with health outcomes? An epidemiological investigation in a UK cohort of 2406 adults.. BMJ Open.

[28] Torres JL, Silva SLA, Lustosa LP (2019). The role of education on the association between disability and depressive symptoms among community-dwelling older adults evidence from Frailty in Brazilian Older People (Fibra) study. Arch Gerontol Geriatr.

[29] Lima-Costa MF, Oliveira C, Macinko J, Marmot M (2012). Socioeconomic inequalities in health in older adults in brazil and england. Am J Public Health.

[30] Torres JL, Vaz CT, Pinheiro LC, Braga LS, Moreira BS, Oliveira C (2023). The relationship between loneliness and healthy ageing indicators in Brazil (ELSI-Brazil) and England (ELSA) sex differences. Public Health.

[31] Golgher A, Coutinho RZ (2020). Life satisfaction in Brazil an exploration of theoretical correlates and age, period and cohort variations using the World Values Survey (1991-2014). Rev Bras Estud Popul.

